# Phenotype of Idiopathic Epilepsy in Great Swiss Mountain Dogs in Germany—A Retrospective Study

**DOI:** 10.3389/fvets.2022.921134

**Published:** 2022-07-12

**Authors:** Theresa Elisabeth Ostermann, Jasmin Nicole Nessler, Hildegard Urankar, Norbert Bachmann, Christel Fechler, Andrea Bathen-Nöthen, Andrea Tipold

**Affiliations:** ^1^Department of Small Animal Medicine and Surgery, University of Veterinary Medicine Hannover, Hannover, Germany; ^2^Great Swiss Mountain Dog Association for Germany e.V., München, Germany; ^3^Veterinary Clinic Dr. med. vet. Andrea Bathen-Nöthen, Cologne, Germany

**Keywords:** idiopathic epilepsy, seizure, phenotype, dog, incidence, breed

## Abstract

Genetic predisposition of idiopathic epilepsy (IE) has been demonstrated in individual breeds. According to the responsible breeding association in Germany, the average incidence of registered Great Swiss Mountain Dogs (GSMDs) with seizures between the years 1999 and 2019 is 2.56%, a genetic predisposition in this breed is suspected. To describe the seizure phenotype and to examine seizure causes, a retrospective, questionnaire-based study was performed. In cooperation with the Swiss Mountain Dog Association of Germany e.V. (SSV e.V.), 114 questionnaires filled in by owners of GSMD displaying seizures and filled in by their respective veterinarians between the years 2005–2021 were evaluated. Seizure characteristics, clinical and further examinations, treatment, treatment responses, and pedigree information were collected. In this study, 94 (83.06%) dogs had IE (suspected genetic epilepsy) confirmed with confidence level TIER 1, 2, or 3. The remaining 20 dogs showed the signs of structural epilepsy, reactive seizures, or epilepsy of unknown cause and were therefore excluded from further analysis. The average age at seizure onset was 28.83 months. Male GSMDs were significantly more often affected by IE than females. The most common seizure type was focal evolving into generalized seizures (64.5%). Seizures often began with vomiting, retching, or salivation. Cluster seizures (CS) (48.9%) and status epilepticus (SE) (37.2%) were observed in a large proportion of dogs. During the observation time, a total of 49 animals (52.13%) died. Out of those, 19 dogs (20.21%) were euthanized in SE or during CS and 14 dogs (14.9%) died spontaneously during CS or SE. The median age at death was 4 years, and the median survival time for the time, when the dog was suffering from seizures, was found to be 18 months. Both occurrence of CS (*p* = 0.0076) and occurrence of SE (*p* = 0.0859) had an impact on survival time. In GSMD, idiopathic epilepsy presents with a severe phenotype with frequently occurring CS and SE. This study could serve as basis for further genetic evaluations as well as to provide individual treatment recommendations.

## Introduction

Epilepsy is one of the most common neurological chronic diseases in humans and dogs (1–3). The prevalence is generally reported to be 0.6–0.75% in the overall canine population and up to 18.3% in certain breeds (1, 4–6). Epilepsy is described as a disorder of the brain, characterized by a persistent predisposition to produce epileptic seizures (7, 8). The definition of seizure etiology has been standardized for veterinary medicine by the IVETF consensus: a distinction is made between structural epilepsy (StE) and idiopathic epilepsy (IE), which in turn both differ from reactive seizures (RS) (7).

Furthermore, according to its etiology, IE is divided into epilepsy of unknown cause, genetic epilepsy, and suspected genetic epilepsy (7), with this study referring to the latter. The basis of suspected genetic epilepsy is formed by familial accumulation, breed prevalence >2%, and/or genealogical analysis (7). In contrast to genetic epilepsy, the identification of a causative gene has not yet been determined (7). Some dog breeds, including a striking number of herding and working dog breeds, are already described to be predisposed to IE (2, 6, 9). It should be noted that a severe clinical manifestation of IE has already been confirmed in herding dogs such as the Australian Shepherd and the Border Collie (10, 11). The Swiss Mountain Dog Association of Germany e.V. (SSV e.V.) recorded 6,179 registered Great Swiss Mountain Dogs (GSMDs) in their database during 1999–2019, of which 158 dogs were registered to have seizures (12). However, not all dog owners provided data about their GSMD, an unknown number of additional dogs with seizures might be suspected. On average, this results in a rate of 2.56% GSMD with seizures and during the years 1999–2019, the frequencies vary from 0.93 to 4.41% (12). This is not equal to the rate of German Great Swiss Mountain Dogs with IE. Based on Sauer-Delhées et al.'s (13) study, the Swiss Kennel Club for Great Swiss Mountain Dogs reported a rate of 2% of dogs with epilepsy between 1999 and 2019 in Switzerland. In contrast to this study, in the Swiss questionnaire-based study, data of 12 GSMD were provided for analysis within less than a year (13). In addition, it was found that there are significant differences regarding heritability, clinical manifestation, seizure semiology, and treatment responses of IE between “geographically distinct populations of the same breed (6)”.

The present questionnaire-based study is therefore dedicated to clarifying the hypothesis, which idiopathic epilepsy in German Great Swiss Mountain Dogs is characterized by a severe phenotype.

## Materials and Methods

### Study Population

This retrospective study was conducted in collaboration with the SSV e.V. This breeding club designed the questionnaire after contacting Diplomates of the European College of Veterinary Neurology and provided questionnaires of Great Swiss Mountain Dogs presenting seizures, collected from 2005 to 2021. Thus, several generations and litters in the population of German GSMD showing seizures, registered in the SSV e.V. and bred within or outside the SSV e.V., were identified, but the exact number of GSMDs with seizures is not known. The SSV e.V. was informed either by breeders or owners of the specific dogs displaying seizures and provided the questionnaires. As a further requirement, clinical and imaging examinations from veterinary practices and clinics were used. These included blood, urine, and cerebrospinal fluid (CSF) examinations, findings of ultrasonography, radiographs, and magnetic resonance imaging (MRI) or computed tomography scans (CT), as well as video recordings of the seizure events. Furthermore, a statement regarding the clinical assessment and evaluation of the further examinations was completed by the treating veterinarian or by the SSV e.V. consulting veterinarian. In addition, based on this information, expert opinions were written by Diplomates of the European College of Veterinary Neurology for the majority of the questionnaires, to determine whether the dogs were suspected or reasonably suspected of having idiopathic or structural epilepsy or reactive seizures, depending on the completeness of the available data. With the help of the patient management system (“easy vet,” VetZGmbH, Isernhagen, Germany) of the Clinic for Small Animals, Foundation of the University of Veterinary Medicine Hannover, additional information on study participants who were patients there themselves could be obtained.

Idiopathic epilepsy was diagnosed according to the International Veterinary Epilepsy Task Force consensus proposal confidence levels (14). Dogs with IE are usually between 6 months and 6 years old at the age of their first seizure and show at least two unprovoked epileptic seizures 24 h apart (14). In addition, physical and neurological examination, minimum database blood test, and urinalysis are without specific findings in the interictal period, which corresponds to the TIER 1 confidence level of the IVETF (2, 14). Based on this, fasting and postprandial bile acids are carried out at the TIER 2 level, as well as MRI of the brain and CSF examination, which should also be unremarkable (14). If electroencephalography (EEG) is also performed, this is referred to as the TIER 3 level (14).

The questionnaire was divided into individual topics and included the following items: signalment of the dog, medical history of the dog, seizure history, age of the dogs at seizure onset, seizure frequency and duration, seizure characteristics, used antiseizure drugs (ASDs), and effect and side effects of the ASDs. Through further contact of the participants with the SSV e.V. or the Clinic for Small Animals of the University of Veterinary Medicine Hannover Foundation, time of death, cause of death, and possible response to therapy could be additionally determined, if these had not already been noted in the questionnaire.

**Figure 1:**
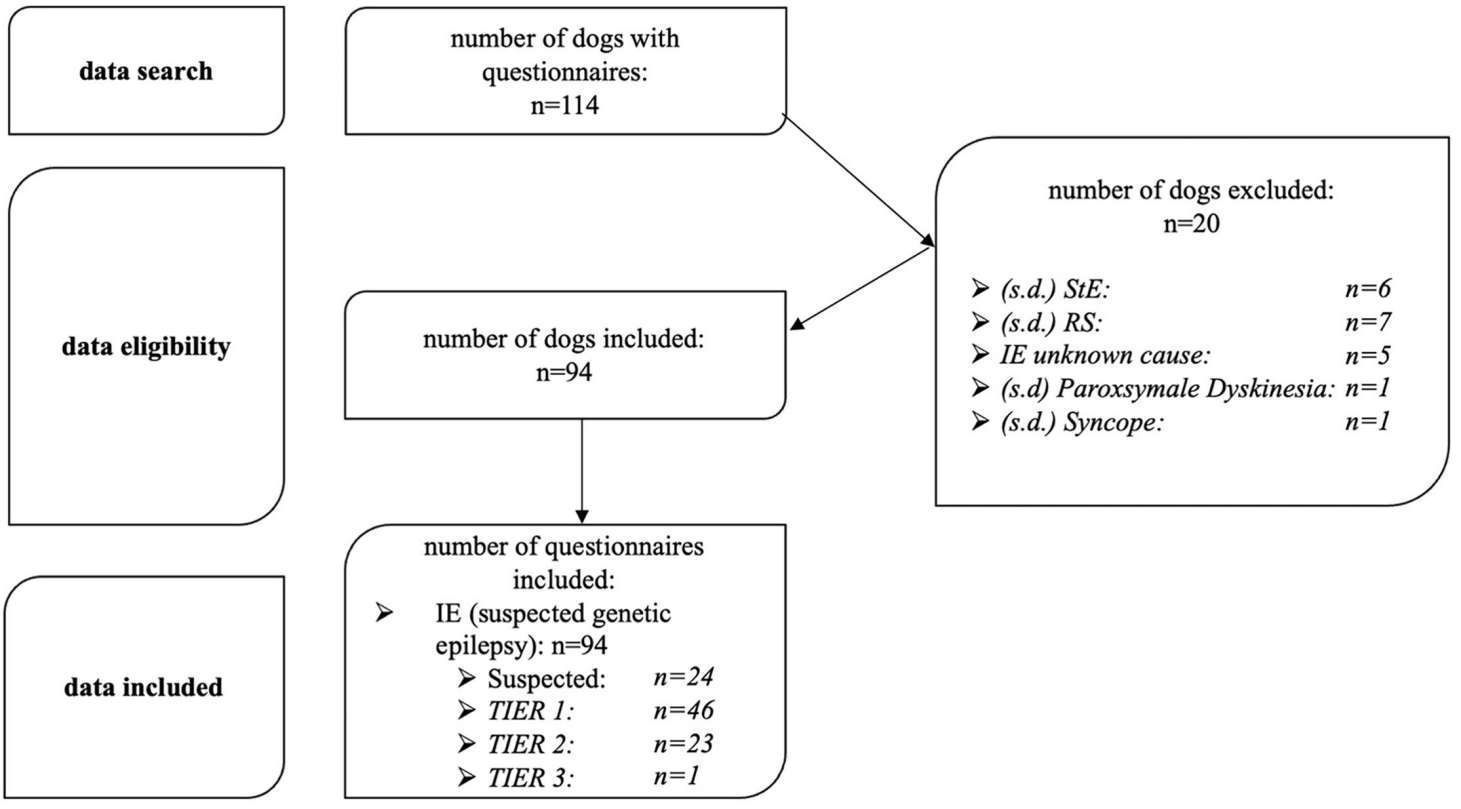
Study protocol for data analysis, inclusion and exclusion criteria. n, number of dogs; StE, structural epilepsy; IE, idiopathic epilepsy; suspected, suspected idiopathic epilepsy; TIER, confidence level (TIER 1,2,3); RS, reactive seizures; s.d., suspected diagnosis.

After the data review of the information collected on each animal, 20 dogs with confirmed or presumed structural brain disease, reactive seizures, syncope, or paroxysmal dyskinesia were excluded from the study (Figure 1).

Of these, 7 dogs had presumed reactive seizures caused by either hypoglycemia, liver disease, electrolyte disorders, or abnormal hematologic findings such as anemia (15). A total of 6 dogs were diagnosed with structural epilepsy based on MRI or CT findings or were suspected to have structural epilepsy based on age older than 6 years at the onset of the first seizure or abnormal findings on interictal neurologic examination without more in-depth diagnostic testing (14). Additionally, 5 dogs were diagnosed with idiopathic epilepsy of unknown cause. These dogs either had their first seizure at an age of <6 months and had no family history of seizures, or atypical seizure characteristics. Animals <6 months of age or >6 years of age at first seizure onset were not included because they did not have the most common age spectrum of seizure onset for IE as defined by the IVETF (7). Exceptions were dogs that have been subjected to further diagnostics in the sense of confidence level TIER 1 or 2 of the IVETF consensus statement and these diagnostics proceeded without any special findings (2 dogs) (14). Furthermore, syncope was suspected in one dog, paroxysmal dyskinesia was suspected in another, and thus, both were excluded from the study (14, 16). Dogs suspected of suffering from IE because of typical seizure characteristics, familial accumulation of the seizure disorder, and required age at first seizure onset between 6 months and 6 years were included (24 dogs; Figure 1).

After applying all inclusion and exclusion criteria, data from 94 study participants with suspected or diagnosed idiopathic epilepsy with suspected genetic origin (confidence levels TIER 1, 2, and 3) could be analyzed (Figure 1).

### Statistical Analysis

The subsequent statistical analysis was performed using Microsoft® Excel 2021 and a commercial statistical software [Statistical Analysis System for Windows SAS®, version 9.4 using the SAS® Enterprise Guide® Version 7.15 Client (SAS Institute Inc., Cary, North Carolina, USA)]. The data to be evaluated were checked for normal distribution using the Shapiro–Wilk and Kolmogorov–Smirnov tests and by visual assessment of individual histograms. Descriptive statistics are provided; in case of a non-significant deviation from the normal distribution, mean values were taken. In contrast, when there was a significant deviation from the normal distribution, the median (m) and minimum and maximum (min–max) were described. Logistic regression was used to assemble correlations between qualitative characteristics, as well as between quantitative and qualitative variables. The Wilcoxon two-sample test was applied to non-normally distributed quantitative data and the Fisher's exact test to qualitative data. A *p*-value of < 0.05 was considered statistically significant and a *p*-value < 0.1 was considered statistically noticeable.

## Results

### Study Population

The participating study population of the present work consists of a total of 94 Great Swiss Mountain Dogs with idiopathic epilepsy. IE was diagnosed or suspected in these dogs according to confidence level 1–3 due to typical seizure characteristics, familial accumulation, and required age at seizure onset between 6 months and 6 years (suspected IE: 24 dogs; TIER 1: 46 dogs; TIER 2: 23 dogs; TIER 3: 1 dog). Overall, MRI and CSF examination were performed for 23 (24.5%) of the dogs (TIER 2 confidence level).

Deviations from the basic population of 94 included dogs in the descriptive analysis result from incomplete questionnaires and were thus evaluated as missing and not interpreted further.

**Figure 2:**
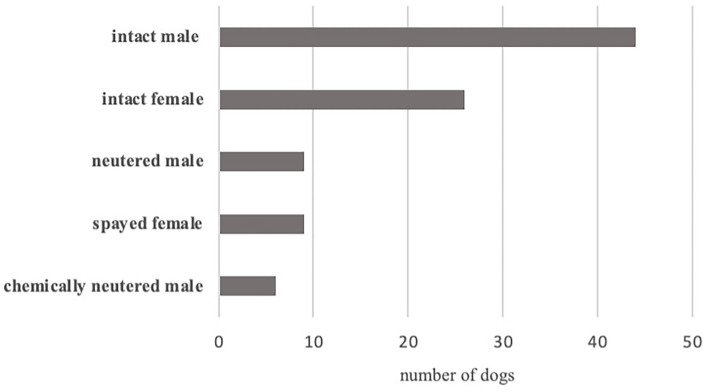
Distribution of sex of Great Swiss Mountain Dogs with idiopathic epilepsy, whole study population.

The gender distribution of GSMD is 37.2% female (*n* = 35) and 62.8% male (*n* = 59). Of these, 9 (9.6%) were spayed female, 26 (27.7%) were intact female, 6 (6.4%) were chemically neutered male, 9 (9.6%) were neutered male, and 44 (46.8%) were intact male (Figure 2). Male GSMDs were found to be significantly more likely to be affected than females in this study population (*p* = 0.0133).

The average weight of the animals is 50.29 kg (range 31–75 kg). The median shoulder height is 69.5 cm (range 52–76 cm). The age of the dogs at seizure onset averages 28.83 months (range 4–79 months). However, neither sex (*p* = 0.6613) nor reproduction status (*p* = 0.1179) showed a significant effect on age at seizure onset.

### Patient History

A history of special events at birth was reported for 14 (15.2%) GSMD. This included information that the dog was the first-born puppy before a caesarian-section was necessary (*n* = 4; 4.4%), that delivery occurred by caesarian-section (*n* = 6; 6.5%), or that stillbirths occurred in the litter (*n* = 2; 2.2%). Pre-existing conditions were mentioned in 28 (30.1%) dogs, these included allergies/food intolerances (*n* = 11; 11.8%), gastrointestinal diseases (*n* = 14; 15.1%), cardiac diseases (*n* = 1; 1.1%), infection-related diseases (*n* = 15; 16.1%), and liver or kidney diseases (*n* = 2; 2.2%).

For 7 (7.5%) dogs, owners reported accidents in the form of a car accident (*n* = 2; 2.2%), contact with an electric fence (*n* = 1; 1.1%), or an unobserved event (*n* = 4; 4.3%).

In 49 (52.69%) dogs, owners were aware of relatives of their dog experiencing epileptic seizures.

About 2 weeks before their dog's first seizure, 5 (5.4%) owners reported an event in terms of anesthesia (*n* = 3; 3.3%), an infectious disease (*n* = 1; 1.1%), or both (*n* = 1; 1.1%) in their animal.

In addition, periods in which the dog presented episodic behavioral changes such as chewing/smacking (*n* = 10; 10.9%), excessive licking (*n* = 6; 5.5%), restlessness (*n* = 3; 3.3%), fly-snapping (*n* = 2; 2.2%), drowsiness (*n* = 2; 2.2%), clinginess (*n* = 1; 1.1%), aggression (*n* = 1; 1.1%), and hallucinations (*n* = 1; 1.1%) were recorded.

### Seizure Characteristics

In more than half of the dogs (*n* = 60; 64.5%), focal epileptic seizures evolving into generalized epileptic seizures were described (Table 1). Focal epileptic seizures started with vomiting (*n* = 39; 43.8%), retching (*n* = 21; 23.6%), hypersalivation (*n* = 19; 21.4%), or head shaking (*n* = 15; 16.9%) (Table 1). Generalized seizures were described in 31 (33.3%) dogs. A total of 5 (5.4%) dogs had a generalized seizure without observing the onset of the seizure. Single focal seizures were shown by 5 (5.5%) dogs (Table 1). Tonic-clonic seizures were described in the majority of GSMD (73 dogs; 90.1%) and tonic seizures in 8 (9.9%) dogs. In addition, autonomic signs during a seizure event were noted in 68 (82.9%) GSMD in the form of hypersalivation (*n* = 61; 74.4%), urination (*n* = 58; 70.7%), or defecation (*n* = 8; 9.8%).

**Table 1:** Seizure characteristics of Great Swiss Mountain Dogs with idiopathic epilepsy (*n* = 93/100%; *n = 91/100%; *n*^•^ = 89/100%).

**Seizure type**	**Number of dogs**	**Percentage**
Focal seizures*	5	5.49
Generalized seizures	31	33.33
Generalized seizures with unknown start	5	5.38
Focal seizures evolving into	60	64.52
generalized seizures
- Hypersalivation^•^	19	21.35
- Vomiting^•^	39	43.82
- Retching^•^	21	23.60
- Head shaking^•^	15	16.85
Cluster seizure	46	48.94
Status epilepticus	35	37.23

n, number of dogs; n*, number of dogs in the category focal seizures, n^•^, number of dogs in the category focal starts.

Prodromal signs were observed in 24 (25.8%) GSMD and manifested in behavior changes such as restlessness (*n* = 18; 19.4%), clinginess to owner (*n* = 8; 8.6%), searching for quiet places (*n* = 4; 4.3%), disorientation (*n* = 2; 2.2%), or aggression (*n* = 1; 1.1%).

The postictal phase lasted for 1 h (median value; range 1–4,320 min) and was reported in 90 dogs. A total of 60 dog owners provided information on the postictal behavioral changes in their GSMD. Of these, 59 GSMD owners described them as follows: disorientation (*n* = 45; 75%), restlessness (*n* = 30; 50%), ataxia (*n* = 21; 35%), clinginess (*n* = 18; 30%), drowsiness (*n* = 15; 25%), polyphagia (*n* = 15; 25%), polydipsia (*n* = 11; 18.3%), aggression (*n* = 10; 16.7%), vocalization (*n* = 10; 16.7%), blindness/running into objects (*n* = 5; 8.3%), or inappetence (*n* = 1; 1.7%).

Seizure triggers of GSMD were reported by 12 (12.9%) patient owners: seizures occurred predominantly after stressful situations, excitement, exertion/intense play, and specific noises.

In most GSMD, seizures usually occurred early in the morning (*n* = 69; 77.5%), during sleep (*n* = 72; 78.26%), or during periods of rest (*n* = 84; 91.3%). In some dogs, seizures occurred during the day (*n* = 55; 61.8%), at night (*n* = 47; 52.8%), in the evening (*n* = 39; 43.8%), or during excitement (*n* = 15; 16.3%). No differences in the triggering situation or time points were described between the first seizure event and the following ones.

### Seizure Frequency, Cluster Seizures, and Status Epilepticus

The median interval between the first and second seizure in the GSMD study population was 30 days (range 1–79 days). The median number of seizures per 6 months was 4 (range 1–35 seizures/6 months). Most frequently, a seizure lasted 3 min (range 0.2–20 min) and was based on the subjective perception of the owner. The median number of seizures before initiation of ASD treatment was 3 seizures (range 1–7 seizures). The age at initiation of therapy with ASDs was 30 months (median value; range 5–82 months). At least one status epilepticus was observed in 35 (37.23%) GSMDs and 46 (48.94%) dogs showed cluster seizures (Table 1). Of these, 11 dogs developed status epilepticus only and 22 dogs displayed cluster seizures only, with 24 dogs experiencing both. A correlation between the occurrence of cluster seizures and status epilepticus was shown in the present GSMD study population (*p* = 0.0052).

However, evidence for a correlation between age at seizure onset (*p* = 0.9719), interval in days between first and second seizure (*p* = 0.9472), number of seizures per 6 months (*p* = 0.5688), and gender (*p* = 0.3710) and occurrence of status epilepticus could not be demonstrated. In addition, age at seizure onset (*p* = 0.4159) or the interval in days between the first and second seizures (*p* = 0.8245) did not significantly affect the occurrence of cluster seizures.

### ASD Treatment

In the study population, continuous ASD treatment was administered to 72 dogs (76.6%). In those cases, 54 (57.4%) dogs were treated with 1 ASD, 17 (18.1%) with 2 ASDs, and one (1%) dog with 3 ASDs. Phenobarbital was administered to 60 (63.8%) GSMD. The last dosage the owners administered to their dogs of continuous phenobarbital application given was 5.8 mg/kg/d (median value; range 1.1–11.3 mg/kg/d) and was reported in 57 dogs. Phenobarbital serum levels were reported in 19 cases (median 20.12 μg/ml; range 5.5–38.7 μg/ml). Imepitoin was used at a median dose of 23.6 mg/kg/d in 17 dogs. Potassium bromide was used at a median dose of 23 mg/kg/d in 14 dogs. Levetiracetam was administered to 4 dogs: of these, 1 dog received pulse protocol, modified from Packer et al. (17), and another dog received levetiracetam after each seizure, but no protocol was specified. The third GSMD was treated with a dose of 95.2 mg/kg/d, and for the fourth dog, only the drug was mentioned. A total of 35 (37.2%) owners reported treating or having their dog treated with diazepam during seizures. In addition, medium chain triglyceride oil was administered in 3 dogs with no indication of the dosage or regimen of use.

The following ASD side effects were observed in 43/62 (69.4%) GSMD: drowsiness (*n* = 18; 29%), polyphagia (*n* = 25; 40.3%), polydipsia (*n* = 22; 35.5%), incoordination/unsteady gait (*n* = 11; 17.7%), or vomiting (*n* = 1; 1.6%). The adverse reactions occurred in 36/57 (63.2%) dogs after phenobarbital administration, in 9/15 (60%) animals after imepitoin administration, and in 7/15 (46.7%) after potassium bromide administration.

Owners reported that 5 (9.4%) dogs became seizure free after ASD treatment. In another 5 (9.4%) dogs, owners could not give exact details about the treatment response. In 27 (50.9%) GSMDs, owners reported a reduction in seizure number, seizure length, or seizure intensity at the time of questionnaire submission. This information corresponds to the subjective feeling of the pet owner, as an exact seizure frequency could not be calculated based on the study design and questionnaire evaluation. According to the owners, 16 (30.2%) GSMDs did not respond to ASD treatment.

Statistically, the age of the GSMD at seizure onset showed no significant effect on the response to therapy (*p* = 0.2617).

### Outcome

A total of 49 (52.13%) GSMDs died in the study population during the study period. Of these, 19 (20.2%) were euthanized in SE or CS, and 14 (14.9%) dogs died spontaneously during SE or CS. Another 3 (3.2%) dogs died, and 6 (6.4%) dogs were euthanized during this period with no indication of cause of death. In 7 (7.5%) dogs, only the time of death was noted, leaving the manner and cause of death unknown. Moreover, 11 of the dead dogs were diagnosed with IE TIER 2 and one dog with TIER 3. In addition, in 45 (47.9%) dogs, the survival status was not described (Table 2). Since there is no explicit question regarding this aspect, it can be assumed that these 45 dogs are still alive.

**Table 2:** Survival rate of the entire study population at the time of evaluation; SE, status epilepticus; CS, cluster seizure.

**Outcome**	**Number of dogs**	**Percentage**
Death, unknown reason	10	10.64
Death during SE or CS	14	14.89
Euthanasia because of SE or CS	19	20.21
Euthanasia, unknown reason	6	6.38
Probable alive	45	47.87

Dogs died with a median age of 48 months (range 10–207 months), with a median survival time, from first seizure to time of death, of 18 months (range 0.5–188 months). Neither age at seizure onset (*p* = 0.2217) nor gender (*p* = 0.3419), number of ASDs applied (*p* = 0.6942) or administration of phenobarbital (*p* = 0.4125) or administration of imepitoin (*p* = 0.1791) were able to significantly influence survival time. In addition, neither gender (*p* = 0.9324) nor reproduction status (*p* = 0.3809) had a significant effect on age at death. However, the occurrence of CS was found to have a significant impact on survival time (*p* = 0.0076), and the occurrence of SE was found to have a statistically noticeable impact on survival time (*p* = 0.0859).

## Discussion

In this study, the phenotype of idiopathic epilepsy in GSMD is described.

In predisposed breeds, the prevalence of IE ranges from 1.25 to 18.3%, and over the years, more and more dog breeds have been added to the predisposition list for idiopathic epilepsy (4, 6, 18–22). The basis for a suspicion of genetic epilepsy is a familial accumulation, a breed prevalence of >2% and/or a genealogical analysis (7). From the mountain dogs, the Bernese Mountain Dog is already on this list (6, 21). The SSV e.V. also became aware of increased numbers of GSMD exhibiting seizures and reported a rate of 2.56% GSMD with seizures, with frequencies varying from 0.93 to 4.41% during the years 1999–2019 (12). More than half of the owners who participated in the present questionnaire-based study had knowledge of their dogs' relatives presenting epileptic seizures.

Based on these findings, a breed predisposition of idiopathic epilepsy is highly suspected in the Great Swiss Mountain Dog and the hypothesis of this study that GSMD predominantly present a severe phenotype of idiopathic epilepsy needs to be investigated.

The statistical analysis of the underlying study population indicates that male GSMDs are significantly more often affected by IE than females. This finding is supported by other studies that have previously demonstrated the same effect in some other breeds, such as the Bernese Mountain Dog (21), Australian Shepherd (11), Finnish Spitz (23), Irish Wolfhound (4), and Golden Retriever (24). The sex distribution of GSMD in the Swiss study by Sauer-Delhées et al. (13), which has a smaller number of participants, records 61.8% male affected animals, mirroring our results.

Another characteristic considered in our study is the age of GSMD at seizure onset, which is on average of 2.4 years. This is slightly lower than the Swiss GSMD of the Swiss Breeders' Association in which the onset of the disease is reported to be 3 years on average (13). However, the time of seizure onset in other herding dog breeds such as the Bernese Mountain Dog was reported to be 2.2 years (21) and for the Border Collie at 2.37 years (10). Those numbers are very similar to our findings and further support our analysis.

A differentiation between primary generalized seizures and focal seizures evolving into generalized seizures is elementary. Focal seizures have a regional origin and are characterized by motor signs, such as facial twitching or head shaking (25, 26). Possible signs can also be attributed to the autonomic nervous system, such as vomiting and salivation, or manifest in behavioral changes and are characterized by their short duration (seconds to minutes) and their frequent transition into a generalized epileptic seizure (7, 25). Primary generalization has been reported as the most frequently occurring seizure type in many dog breeds in the older literature (6, 21, 24). After the implementation of the described differentiation, focal seizures with secondary generalization are now referred to as the dominant seizure type in dogs with idiopathic epilepsy (7). Furthermore, the described seizure type is also reported in most dog breeds, such as the Poodle with 60% (27) and the Border Collie with 78% (10). In this study, focal seizures, which generalize secondarily, were described in more than half of the dogs (64.5%) and presented as vomiting (43.8%), retching (23.6%), hypersalivation (21.4%), or head shaking (16.9%). Likewise, Sauer-Delhées et al. (13) recorded focal motor seizures with secondary generalization in more than 50% (7/12) of the cases in the Swiss GSMD population. However, in their study, characteristics such as vomiting shortly before a seizure were assigned to autonomic signs and not to the focal onset (13), which affects the comparability.

Administration of ASDs forms the basis of IE therapy (28). In a study by Berendt et al. (29), however, no statistically significant differences in age at death or number of years with epilepsy were found between drug-treated and untreated dogs. However, in a previously published manuscript, an increase in seizure frequency without long-term therapy was detected (30). Considering the treatment response to ASDs in our study, seizure freedom was achieved in only 5 (9.4%) dogs. Seizure freedom, respectively, a positive treatment response in studies, is defined as a seizure-free period equal to more than three times the length of the longest interictal period prior to initiation of therapy and lasting at least 3 months (31). Furthermore, 27 (50.9%) owners of German GSMD reported a reduction in seizure number, seizure length, or seizure intensity after the administration of ASDs at the time of questionnaire completion compared to the start of ASD administration. To determine the percentage of seizure frequency reduction and time frame of seizure freedom, the baseline seizure frequency should be considered, and the exact seizure frequency must be calculated (31). Such data were not sufficiently reported in the questionnaire of this study. However, data on seizure reduction and seizure freedom in canine epilepsy correspond to the subjective perception of the animal owners (10, 11, 13). According to owners in our GSMD study population, 16 (30.2%) dogs responded inadequately to ASD treatment. Considering subjective perception, the aspects of remission (9.4%) and seizure reduction (50.9%) of our German GSMD are reflected in similar studies (10, 11, 13). Compared to other breeds, according to Packer et al. (32), the Border Collie was the breed least likely to go into remission (0%) or achieve a reduction in seizure frequency of ≥50% (40%) (32). However, in Hülsmeyer et al. (10), the remission rate of Border Collies was 18%. In Australian Shepherds, a remission rate of 13% was reported (11). In the Swiss study of GSMD with IE, 33.3% of animals (4/12) showed a reduced seizure frequency with therapy; however, the remaining 66.7% (8/12) did not respond adequately to antiseizure drugs (13).

An older age at seizure onset seems to be associated with an increased likelihood of achieving remission or reduction in seizure frequency by ≥50% (32). In the study population of Australian Shepherds by Weissl et al. (11), a relationship between age at seizure onset and seizure control was also detected. However, in this study, age of GSMD at seizure onset did not reveal a statistically significant effect on response to treatment. This was in line with the results of evaluations of German Border Collies with IE (10). Consequently, the multifactorial influences on treatment response and drug resistance are presented and mirrored in this study, influences, which remain largely unknown despite years of research into the underlying mechanisms (33).

Complementarily, under the influencing risk factors for achieving remission, the occurrence of cluster seizures should be considered (32). In this study, 35 (37.23%) GSMDs suffered at least one status epilepticus, 46 (48.94%) dogs showed cluster seizures, and a correlation between the occurrence of cluster seizures and status epilepticus could be established. Similar results were also detected in the following studies: Sauer-Delhées et al. (13) determined the occurrence of status epilepticus in 41.6% of GSMD in Switzerland and 50% suffered cluster seizures. In a study of Belgian Shepherds with IE, serial seizures were reported in one-third of the dogs (34). Cluster seizures were described in 68% of Australian Shepherds with IE and status epilepticus in 60% (11). In the German study by Hülsmeyer et al. (10), a total of 94% of all included Border Collies experienced at least one episode of cluster seizures and 60% of all dogs experienced at least one episode of status epilepticus.

Furthermore, in our study, neither the influence of age at first seizure nor the time in days between the first and second seizure proved to be statistically significant on the occurrence of cluster seizures or status epilepticus. Similarly, no statistically significant relationship was found between sex and the occurrence of status epilepticus. The evaluation of the Australian Shepherd study revealed the same findings, and the severity of the clinical course, meaning the occurrence of serial seizures or a status epilepticus, was also independent of the age at seizure onset and of gender (11).

The occurrence of cluster seizures and status epilepticus in contrast to single, short seizures does not only have an impact on the severity of the clinical course, but also on the decision to euthanize the diseased dog: approximately two-thirds of dogs with epilepsy are euthanized due to status epilepticus or cluster seizures (35). Studies found that the occurrence of cluster seizures is associated with a shorter survival time (35), as well as a higher possibility of euthanasia (36). In addition, dogs that suffer from serial seizures have a lower chance of achieving seizure freedom (32). It should also be noticed that patients who did not receive therapy had a significantly increased risk of being euthanized (29). In a study of “Risk Factors for Survival of Dogs with Epilepsy”, death or euthanasia was directly associated with epilepsy in 52% of dogs, with a significant proportion experiencing pre-existing cluster seizures, status epilepticus, or both (36).

Overall, in the study population presented here, 52.13% (49/94) of GSMDs died during the study time. About 35.1% (33/94) dogs were euthanized or died spontaneously in direct association with IE. Proportionately, 20.2% (19/94) were euthanized in status epilepticus or during cluster seizures. About 14.9% (14/94) of dogs died spontaneously during status epilepticus or cluster seizures.

In comparison, in the Swiss GSMD study, 25% (5/20) of the affected animals died due to seizures (13). This result can be attributed to the much shorter research time in contrast to the discussed study. In Irish Wolfhounds with IE, the lethality was reported to be 60.3% (76/126) (4). For comparability with our work and others, a mortality of 52% (76/146) can be calculated here from the total study population (4). In a Border Collie study, 47% (23/49) of the participating dogs had died at the time of evaluation: 74% (17/23) of the animals were euthanized due to IE and two dogs died in SE (10). Again, a mortality of 34.7% (17/49) can be calculated from the total study population of Border Collies (10). Regarding Petit Basset Griffon Vendéen with epilepsy, 45/471 dogs died in a study population, of which 13.3% (6/45) died from epilepsy itself (20). However, when converted to the total study population, consisting of 471 Petit Basset Griffon Vendéen, only 1.3% (6/471) died as a direct result of epilepsy (20). In the Australian Shepherd, death due to IE was reported in 28% (14/50) (11).

In our German GSMD with IE, the median age at death was 4 years with a median survival time from first seizure to time of death of 1.5 years. This outcome was also shown by Heske et al. (5) in a cohort study of epilepsy, in which the median survival time after diagnosis was reported to be 1.5 years. In another study on “Premature Death, Risk Factors, and Life Patterns in Dogs with Epilepsy”, the median age of several breeds of dogs at death was found to be 7 years and the survival time was found to be 2.3 years (29). The median age of Australian Shepherds at death was 3.1 years; there was no calculation of survival time (11). German Border Collies with IE, on the other hand, were slightly older (5.17 years) than Australian Shepherds or GSMD at time of death; the median survival time (2.07 years) of Border Collies was similar to our results (10). Such a short survival time in GSMD with IE should be highly considered to develop further studies on new treatment schemes.

Statistically, no significant influence on survival time by age at first seizure, sex, number of antiseizure drugs, or administration of phenobarbital or imepitoin was found in the study discussed here. In contrast, Heske et al. (5) and Berendt et al. (29) found that females lived longer than males after a diagnosis of epilepsy, although this refers to a mixed dog breed population. Also, in the mixed population of dogs with IE, a higher age at diagnosis correlated with a shorter survival time (5). On the other hand, in a study on Border Collies, comparable to our study, there was no significant correlation between survival time and sex, reproduction status, or number of drugs administered (10).

In addition, in German GSMD, neither sex nor reproduction status had a significant effect on age at death. This was also confirmed by the study on German Border Collies (10). Regardless of this, this study was able to highlight that the occurrence of cluster seizures has a significant impact on survival time and the occurrence of status epilepticus has a statistically noticeable impact on survival time. Both cluster seizures and status epilepticus have been reported as risk factors for survival in dogs with epilepsy in previous studies (29, 35, 37), what has also been demonstrated in Border Collies (10). Treatment of these severe forms of seizures in GSMD such as a new concept of a pulse therapy could be considered (17).

Furthermore, a retrospective study of 407 dogs with epilepsy found an association between cluster seizure frequency and euthanasia, as significantly more dogs with frequently occurring CS were euthanized compared to dogs with less frequently occurring CS (35). Owners struggle with enduring the occurrence of cluster seizures or status epilepticus in their dog (29, 38). This not only significantly affects the quality of life of pet owners, but also the decision to euthanize their dog (36, 39). It can be suspected that owners of Great Swiss Mountain dogs with recurrent CS and SE have a similar reduced quality of life and fear that their pet is suffering, what leads to early decision for euthanasia.

In 52.69% (49/94) of GSMD relatives displayed seizures, strengthening the assumption of the genetic component already supported by the SSV e.V. surveys with a rate of 2.56% of dogs with seizures in 1999–2019. Sauer-Delhées et al. (13) stated that 28% (36/128) of all GSMD included in the study had a history of epilepsy in the family. Furthermore, in 45% (9/20) of the 20 dogs with seizures, seizures also occurred in family members (13). In Border Collies, a common ancestor could be identified in 29 affected dogs, “strengthening the suspicion of a genetic background of idiopathic epilepsy in Border Collies” (10). In the Australian Shepherd study, a pedigree of 42 IE-affected dogs was established, revealing a common ancestor of 29 affected Australian Shepherds in two subpopulations and an affinity for cluster seizures in littermates, full siblings, or half siblings (11). Pedigree analysis of Hungarian Magyar Vizsla revealed that all affected dogs in the study were traced to a common sire (40). Nevertheless, the identification of a causative gene has not been successful in the above-mentioned breeds (10, 11, 13, 21, 40, 41) and was not part of the objective in the discussed study.

A limitation of this study is its retrospective design. In some cases, not all data were available. In addition, the suspected diagnosis IE was based on the questionnaires and additional information from the treating veterinarians, without MRI and CSF examination (TIER 1). However, this influence was minimized by re-examining the dogs' data. In addition, the questionnaire contained standardized questions, and all free-text responses were compared with medical records.

In conclusion, the hypothesis that German Great Swiss Mountain Dogs show a severe clinical course of idiopathic epilepsy similar to other breeds such as the Australian Shepherd or Border Collie (6, 10, 11) can be confirmed in this study because of substantial percentages of dogs with status epilepticus and cluster seizures, as well as a high mortality rate. The current results can serve as a basis for genetic evaluations to identify a causative genetic defect, as well as to provide individual therapy recommendations, such as modified medication management during preictal phases, adding a second or third drug in case of an insufficient drug response and to raise awareness among GSMD owners, breeders, and the veterinary community.

## Data Availability Statement

The datasets used and analyzed during the current study are available from the corresponding author on reasonable request.

## Ethics Statement

The study was conducted in accordance with the Animal Welfare Act by following the ethical and data protection guidelines of the University of Veterinary Medicine Hannover. The owners gave their informed consent to the anonymously evaluated scientific analysis of the data when filling out the questionnaire.

## Author Contributions

HU, NB, AT, and JN were responsible for the conception of the study. AT, AB-N, HU, NB, and CF performed the data acquisition. TO performed the statistical analysis, data analysis, and manuscript writing. AT supervised the data collection and manuscript editing. All authors contributed to the article and approved the submitted version.

## Funding

This Open Access publication was funded by the Deutsche Forschungsgemeinschaft (DFG, German Research Foundation) within the programme LE 824/10-1 Open Access Publication Costs, and University of Veterinary Medicine Hannover, Foundation.

## Conflict of Interest

The authors declare that the research was conducted in the absence of any commercial or financial relationships that could be construed as a potential conflict of interest. The handling editor AF declared a past co-authorship with the author AT.

## Publisher's Note

All claims expressed in this article are solely those of the authors and do not necessarily represent those of their affiliated organizations, or those of the publisher, the editors and the reviewers. Any product that may be evaluated in this article, or claim that may be made by its manufacturer, is not guaranteed or endorsed by the publisher.

## Abbreviations

ASD: Antiseizure drug
CS: Cluster seizure
CSF: Cerebrospinal fluid
CT: Computed tomography scans
EEG: Electroencephalography
GSMD: Great Swiss Mountain Dog
IE: Idiopathic epilepsy
IVETF: International Veterinary Epilepsy Task Force
μl: Microliter
mg: Milligram
ml: Milliliter
MRI: Magnetic resonance imaging
n: Number of dogs
RS: Reactive seizures
SE: Status epilepticus
StE: Structural epilepsy
SSV e.V.: Swiss Mountain Dog Association of Germany e.V.
TIER 1-2: Confidence level for the diagnosis of IE.
